# Adolescent gender differences in the determinants of tobacco smoking: a cross sectional survey among high school students in São Paulo

**DOI:** 10.1186/1471-2458-10-748

**Published:** 2010-12-03

**Authors:** Zila M Sanchez, Emerita S Opaleye, Silvia S Martins, Jasjit S Ahluwalia, Ana R Noto

**Affiliations:** 1 Brazilian Center of Information on Psychotropic Drugs (CEBRID), Psychobiology Department of the Universidade Federal de São Paulo, Brazil; 2 Department of Mental Health, Johns Hopkins Bloomberg School of Public Health, Baltimore, USA; 3 Center for Health Equity at the University of Minnesota, Minneapolis, USA

## Abstract

**Background:**

Diverse psychosocial factors have been associated with the use of cigarettes by adolescents. We investigated gender differences in tobacco smoking, and factors correlated with smoking among boys and girls.

**Methods:**

Data was collected on recent cigarette smoking (CS) and related factors, with a focus on religious beliefs, leisure activities, family structure, relationships and parental monitoring from 2,691 private school-attending youths from 28 schools in São Paulo, Brazil, selected via probability sampling. Estimates were derived via weighted hierarchical logistic regression models.

**Results:**

There was no difference in the prevalence of recent cigarette smoking between boys and girls (14.2%). Older age (_a_OR_boys _= 1.71[1.33-2.21]; _a_OR_girls _= 1.73[1.35-2.23]), second-hand smoke exposure at home (_a_OR_boys _= 1.52[1.00-2.29]; _a_OR_girls _= 1.74[1.13-2.68]) and not having a religion (_a_OR_boys _= 1.99[1.41-2.81]; _a_OR_girls _= 1.78[1.14-2.78]) were associated with CS in boys and girls. Adolescents who went out often at night were more likely to be tobacco smokers (_a_OR_boys _= 8.82[3.96-19.67]; _a_OR_girls _= 14.20[6.64-30.37]). For girls, data suggest that CS was also associated with a lack of parental attention and care (_a_OR_girls _= 4.37[1.19-16.04]) and no participation in youth religious activities (_a_OR_girls _= 2.76[1.49-5.12]). For boys, CS was additionally associated with the loss of one or both parents (_a_OR_boys _= 3.75[1.78-7.85]).

**Conclusions:**

Although older age, living with smokers at home and lack of religion were common contributing factors to cigarette smoking among all adolescents, girls were influenced to a greater degree by family relationships and religiosity than boys. The study results may be materially important to the development of prevention programs that influence determinants connected to gender and the implementation of single-core models of prevention; gender differences must be considered in order to reduce adolescent CS.

## Background

Tobacco smoking, the main cause of preventable morbidity and death worldwide, is a behavior that starts in adolescence for 90% of the adults who self-report smoking. Serious consequences of smoking, such as cancer and cardiovascular disease, are known to occur later in life [1]. Studies have indicated to various smoking-associated factors in adolescence such as depression, suicidal ideation [2] and the abuse of other substances [3].

In Europe, the prevalence of past month use of cigarettes among high school students was remarkably different between genders, varying from 15% of boys in Iceland to 48% of girls in Austria, with a large number of countries showing higher cigarette consumption among girls [4]. Although there has been a recent decrease in tobacco consumption among high school students in the United States (from 28.3% to 12.6%), boys in older age categories did have slightly higher tobacco consumption [5]. In Brazil, studies conducted among middle and high school students revealed a 10-16% prevalence of cigarette consumption in the last month, with gender differences in different cities. Like the U.S., there has been a decrease in regular tobacco use over the last ten years [6,7].

Diverse psychosocial factors have been associated with the use of cigarettes by adolescents; including: peer pressure, poor parental supervision or familial relationships [8-10], smoking among parents, siblings or partners [11], leisure activities, especially those that involve the company of friends such as nightlife and aspects of religiosity [12].

Evidence from North America suggests that there are gender differences in factors associated with smoking. Boys with strong religious involvement showed slower progression from experimentation to regular smoking. Meanwhile, a positive familial relationship, including care and family activities, protected girls from the first use of tobacco [13]. Studies based on developmental theories suggest that boys and girls may perceive and react to familial influence in different ways and thereby are exposed to different risk factors for drug consumption [14]. These latter studies also suggest the need for gender-specific prevention programs.

Few studies have investigated the relationship between these factors. Recent studies suggest the need to concurrently evaluate the impact of religious and familial themes on drug consumption by adolescents because these are integrated issues with mutual influence [15-17]. Other studies also suggest that familial factors are modified by leisure activities or partner influence when evaluated with regression models [18,19].

This study aims to verify the association among familial factors, religious factors and leisure time activities with smoking by gender among adolescent private school students in Sao Paulo, Brazil, based on the Social Development Model [20]. The Social Development Model presupposes theories of control and social learning, constituting a picture of pro and antisocial behaviors [21,22]. According to this model, adolescents tend to engage in antisocial behaviors when they develop bonds with groups with antisocial beliefs (e.g., drug-using peers) and vice-versa. In addition, external factors such as family relationships and parental monitoring may interfere in the process of bonding with bad behavior peers [21,22].

The relationship between psychosocial factors and smoking may vary by culture [13,14]. The majority of studies have been conducted in North American and European populations [23-25], and studies focusing on Latin American, Asian and African populations are rare. This knowledge gap calls for studies in these cultures.

This study aims to examine gender differences in familial, religious and leisure factors in a sample of adolescents in Sao Paulo, the largest city in Latin America.

## Methods

### Sampling

The study was designed to select a representative sample of high school students at private schools in São Paulo, Brazil, by a two step randomization process. The city has 578 private high schools, and 28 were included in this study. The sample size was set for a maximum 10% relative error and a 95% confidence interval (95% CI). In the first step, the schools were stratified according to the average income of the neighborhood in which they were located. As a second step of randomization, the sample was selected by clusters (classes). All the students in each selected class were invited to complete the questionnaires. The process generated a final sample of 2691 high school students. The response rate among the students invited to participate was 99.4% (only 16 adolescents refused to participate in the study). The complex survey design took into account the stratum (neighborhood), the cluster, the expansion weight and the probability of drawing the student.

### Data collection

Data was collected by a paper and pencil self-report instrument with closed questions based on a World Health Organization instrument [26] and the European School Survey Project on Alcohol and Other Drugs (ESPAD) questionnaire [4], adapted to the Brazilian culture (see Additional file 1). In addition to questions on history of smoking and other substance use patterns, the instrument collected information on the students' socioeconomic status (SES), religious behavior, leisure activities and family functioning and structure.

A database interface was developed to detect typing errors and evaluate the internal consistency of the answers. We also included one question on the use of a fictitious drug, and the students who gave a positive answer (7 cases-0.2%) were excluded.

### Measures

#### Outcome variable

The outcome of interest was cigarette smoking (CS), specifically cigarettes only, and use was defined as at least once in the last month.

#### Independent variable

Sociodemographic variables selected for this study were age, monthly school tuition (obtained directly from schools' principals) and economic scale [27], which considered the educational level of the head of the household, possession of household goods and number of housekeepers. This scale categorized students into groups labeled from A to E (where A was the highest economic strata). Monthly tuition fees were subdivided into quartiles: US$ 100-190, US$ 191-260, US$ 261-480 and US$ 481-1300.

The following leisure behaviors were evaluated according to frequency during the month preceding the survey: "going out to bars and parties with friends at night," "hanging out with friends at parks and shopping malls," "playing videogames," "reading books on your own initiative," "using the internet for fun," "participating in artistic activities such as theater, singing, dancing and others" and "participating in volunteer work." All items included the following frequency responses: *almost every day, at least once a week, one to three times a month *or *never*.

A similarly structured question investigated the religiosity of the students in the past month: "voluntary participation in collective prayer," "youth meetings" and "artistic activities within a religious group." We added one question about "having a religion" (yes or no) and the "intensity of their belief in God or a higher power" for which possible answers ranged from *none at all *to *very much indeed*.

There were questions on family structure, such as the "marital status of his/her parents." We evaluated parental monitoring and parental relationship through questions on the definition of "rules inside" and "outside" the house, "attention and/or care," "provision of money," "meals eaten together," "conversations about school," "who do they go out with," "where do they go," "praise" and "conversations lasting at least 10 minutes." In this module, we examined the frequency of each of these behaviors during the last month. The possible frequency answers were *always, often, sometimes, rarely *and *never*. Finally, we obtained data on "tobacco smoking among the family members with whom the respondents lived".

### Data analysis

Initially, we examined the data by bivariate analyses with a level of significance of 5%, and then, we performed hierarchical logistic regressions for complex samples and included all variables used in the bivariate analyses [28].

Analyses were conducted with data that were weighted to correct for unequal probabilities of selection into the sample. The outcome variable of interest was cigarette smoking (CS) during the past month. The independent variables included sociodemographic factors and behaviors related to leisure, religion and family. Results are presented as weighted proportions (wgt%), crude Odds Ratios (_c_ORs), adjusted Odds Ratios (_a_ORs) and p-values. Hierarchical regressions were developed separately for boys and girls. First, the sociodemographic variables were introduced (Step 1); next, the family-related variables (Step 2) and the variables related with religiosity (Step 3) were added; lastly, the variables related with leisure were added (Step 4). We opted to build a hierarchical logistic regression model because we used 27 variables with an average of 4 answer categories (3 sociodemographic variables, 12 familial variables, 5 religiosity variables and 7 leisure variables). We used the complex samples module of the SPSS Version 17 software to perform the calculations.

### Ethical aspects

The questionnaire did not include any information that could be used to identify the students. This study was approved by UNIFESP's Research Ethics Committee (#0930/07).

## Results

We received 2691 questionnaires, 11 questionnaires had missing answers, so 2680 were considered valid for the analysis of recent CS. 51.9% of the students were female (95%CI 48.9-54.9), 95.5% of higher socioeconomic status (SES A and B; 95%CI 97.4-93.7) and with 16.0 years-old in average (95%CI 15.9-16.1). Fourteen percent of the students reported smoking during the 30 days prior to the study. Table 1 shows the distribution of this behavior by gender. No differences were found in the prevalence of past-month use; and age of onset among boys and girls. In most cases, recent CS occurred between one and five days during the previous month. Both genders in the study usually started to smoke when they were 14 years old.

**Table 1:** Prevalence of past-month tobacco smoking by gender among students in private high schools (n = 2680)

Tobacco Consumption	Boys		Girls		Total		p
	**% (95%CI)**	**Unweighted count**	**% (IC 95%)**	**Unweighted count**	**% (IC 95%)**	**Unweighted count**	
**Past-month use**	14.3 (11.0-18.4)	180	14.0 (10.2-19.0)	207	14.2 (11.5-17.4)	387	0.76
**1 to 5 days**	8.5 (5.9-12.1)	114	8.0 (6.1-10.6)	117	8.3 (6.5-10.4)	231	
**6 to 19 days**	2.5 (1.5-4.0)	29	1.8 (1.0-3.4)	31	2.1 (1.4-3.2)	60	
**20 days or more**	3.4 (1.7-6.4)	37	4.1 (2.6-6.5)	59	3.8 (2.6-5.5)	96	
							
	**Boys**		**Girls**		**Total**		
**Average age of onset**	14.02 (13.69-14.35)		13.98 (13.61-14.35)		14.00 (13.67-14.32)		0.20

Different factors associated with recent CS were considered for the independent logistic regressions models for boys and for girls. Table 2 presents the distribution of variables that remained in the final logistic regression model for each gender as well as the bivariate analyses expressed in terms of _c_OR.

**Table 2:** Significant variables in the final logistic regression model and crude odds for boys (n = 1227) and for girls (n = 1418).

	Recent tobacco use					
Variables for boys	No		Yes		Total	crudeOR [95%IC]
	n (wgt%)	SE	n (wgt%)	SE		
***Monthly tuition *Over US$ 480**	145 (13.8)	6.6	38 (21.8)	9.9	183	3.18 [1.31-7.72]
**US$ 261-US$ 480**	280 (24.5)	8.2	48 (28.0)	9.6	328	2.29 [0.87-6.08]
**US$ 191-US$ 260**	374 (43.0)	10.5	72 (40.8)	11.9	446	1.90 [0.68-5.32]
**Up to US$ 190**	248 (18.8)	7.2	22 (9.4)	4.8	270	Reference
	1047 (100.0)		180 (100.0)		1227	
***Someone you live with smokes ***						
**Yes**	297 (29.1)	1.6	79 (44.2)	3.2	376	1.93 [1.49-2.51]
**No**	731 (70.9)	1.6	98 (55.8)	3.2	829	Reference
	1028 (100.0)		177 (100.0)		1205	
***Parental marital status***						
**One or both died**	36 (3.0)	0.5	14 (9.7)	3.4	50	3.77 [1.45-9.85]
**Don't live together**	237(25.3)	2.0	45 (28.9)	3.7	282	1.34 [0.90-1.97]
**Live together**	761 (71.7)	1.0	117 (61.4)	5.3	878	Reference
	1034 (100.0)		176 (100.0)		1210	
***Do you have a religion?***						
**No**	160 (15.7)	1.3	51 (31.7)	5.2	211	2.49 [1.45-4.29]
**Yes**	881(84.3)	1.3	129 (68.3)	5.2	1010	Reference
	1041 (100.0)		180 (100.0)		1221	
***Goes out with friends at night***						
**At least once a week**	472 (47.9)	2.3	144 (83.1)	3.6	616	11.91 [5,25-27.01]
**1 to 3 times a month**	376 (35.5)	2.2	30 (14.5)	3.2	406	2.81 [1.26-6.25]
**Not even once**	193 (16.5)	1.7	6 (2.4)	1.0	199	Reference
	1041 (100.0)		180 (100.0)		1221	
**Variables for girls**						
***Someone you live with smokes***						
**Yes**	351 (29.6)	1.7	101 (47.5)	3.7	452	2.15 [1.56-2.95]
**No**	846 (70.4)	1.7	105 (52.5)	3.7	951	Reference
	1197 (100.0)		206 (100.0)		1403	
***I can easily get attention and care from my parents***						
**Never**	17 (1.4)	0.3	8 (3.4)	0.8	25	2.72 [1.15-6.46]
**Sometimes/rarely**	282 (21.5)	1.2	59 (28.2)	3.1	341	1.48 [1.10-1.99]
**Always/often**	914 (77.1)	1.3	138 (68.4)	3.7	1052	Reference
	1213 (100.0)		205 (100.0)		1418	
***Voluntary participation in religious youth meeting***						
**Not even once**	872 (72.2)	2.4	175 (87.4)	3.1	1047	3.64 [2.06-6.43]
**1 to 3 times a month**	116 (9.3)	0.9	16 (6.4)	2.1	132	2.07 [0.94-4.58]
**At least once a week**	219 (18.5)	2.0	15 (6.2)	1.6	234	Reference
	1207 (100.0)		206 (100.0)		1413	
***Do you have a religion?***						
**No**	123 (10.4)	1.5	39 (18.2)	4.1	162	1.92 [1.28-2.87]
**Yes**	1084 (89.6)	1.5	168 (81.8)	4.1	1252	Reference
	1207(100.0)		207(100.0)		1414	
***Goes out with friends at night***						
**At least once a week**	534 (46.1)	2.3	154 (75.8)	3.2	688	13.43 [6.00-30.03]
**1 to 3 times a month**	453(34.6)	1.9	47 (21.9)	2.9	500	5.16 [2.33-11.42]
**Not even once**	233 (19.3)	1.9	6 (2.4)	0.9	229	Reference
	1210 (100.0)		207 (100.0)		1417	

### Factors associated to CS for boys

For boys, tuition costs and age were the only two sociodemographic factors associated with CS **(**Table 3). Older age remained associated with tobacco smoking in all steps of the analysis, whereas there were no significant differences between the ORs with the inclusion of familial, religiosity and leisure variables (_a_ORs ranged from 1.71 to 1.84). However, tuition costs became non-significant when leisure variables were inserted in the model.

**Table 3:** Results of the hierarchical logistic regression for factors associated with recent tobacco smoking among boys in private high schools (n = 1227)

	step 1	R^2^		step 2	R^2^	ΔR^2^	step 3	R^2^	ΔR^2^	step 4	R2	ΔR2
		0.09			0.13	0.04		0.15	0.02		0.23	0.08
**Parameter**	**OR**	**CI 95%**	**p**	**OR**	**CI 95%**	**p**	**OR**	**CI 95%**	**p**	**OR**	**CI 95%**	**p**
***Age***	1.84	(1.43-2.36)	0.00	1.82	(1.39-2.38)	0.00	1.78	(1.39-2.23)	0.00	1.71	(1.33-2.21)	0.00
***Tuition***												
**Over US$ 480**	3.54	(1.53-8.14)	0.00	3.70	(1.61-8.51)	0.00	3.61	(1.59-8.21)	0.00	2.29	(0.92-5.71)	0.07
**US$ 261-US$ 480**	2.29	(0.92-5.71)	0.07	2.43	(0.96-6.14)	0.06	2.52	(1.00-6.36)	0.05	2.02	(0.75-5.49)	0.15
**US$ 191-US$ 260**	1.99	(0.74-5.34)	0.16	2.08	(0.77-5.61)	0.14	1.99	(0.76-5.24)	0.15	1.65	(0.57-4.75)	0.33
**Up to US$ 190**	1.00	.	.	1.00	.	.	1.00	.	.	1.00	.	.
***Someone you live with smokes***												
**Yes**				1.68	(1.25-2.26)	0.00	1.68	(1.25-2.27)	0.00	1.52	(1.00-2.29)	0.05
**No**				1.00	.	.	1.00	.	.	1.00	.	.
***Parental marital status***												
**One or both died**				3.56	(1.53-8.31)	0.00	3.19	(1.53-6.65)	0.00	3.75	(1.78-7.85)	0.00
**Don't live together**				1.20	(0.76-1.90)	0.40	1.15	(0.70-1.84)	0.55	1.20	(0.79-1.82)	0.38
**Live together**				1.00	.	.	1.00	.	.	1.00	.	.
***Do you have a religion***												
**No**							2,22	(1.55-3.17)	0.00	1.99	(1.41-2.81)	0.00
**Yes**							1.00	.	.	1.00	.	.
***Goes out with friends at night***												
**At least once a week**										8.82	(3.96-19.67)	0.00
**1 to 3 times a month**										2.46	(1.02-5.92)	0.04
**Not even once**										1.00	.	.

For boys, the two familial factors associated with CS were death of one or both parents and exposure to tobacco inside the home. Those whose parents lived together served as the reference group, and boys whose parents were deceased (one or both) were 3.7-fold (95% CI = 1.78-7.85, p < 0.001) more likely to have smoked. There was no difference in the likelihood of CS between the group of boys whose parents lived apart and the group of boys whose parents lived together. Adolescents in the CS group were 70% more likely to be living with someone who smoked. The strength of this association, although it remained significant (_a_OR = 1.52, 95% CI = 1.00-2.29, p = 0.05), was diminished when leisure activities were added to the model. When religious aspects were added, boys who declared not to have a religion were twice as likely to smoke as those who reported having a religion (_a_OR = 1.99, 95% CI = 1.41-2.81, p < 0.001). Nevertheless, factors that could have demonstrated the influence of religious beliefs and the importance given to religion were not significantly associated with CS.

Regarding leisure activities, the only factor associated with CS was frequency of nightlife gatherings with friends. Night-out episodes with friends for shows, night clubs and pubs at least once a week was associated with a nine-fold likelihood of CS during the last month (_a_OR = 8.82, 95%CI = 3.96-19.67, p < 0.001).

### Associated factors to CS for girls

In the hierarchic regression model for girls (Table 4), age, second-hand smoking exposure at home, having a religion and nightlife were associated with CS. For girls, age was the only sociodemographic factor associated with CS. With each increasing year of age, there was an incremental 1.73-fold (95% CI = 1.35-2.23; p < 0.001) increase in CS, similar to what was observed for boys. The familial factor most associated with CS was self-reported frequency with which adolescents received attention and care from their parents. Girls who reported having never received attention and care from their parents were four times more likely to smoke in the last month than girls who described receiving frequent attention from their parents (_a_OR = 4.37, 95% CI = 1.19-16.00, p = 0.03). Similar to boys, living with someone who smoked increased the likelihood of CS for girls by more than 70% (_a_OR = 1.74, 95% CI = 1.13-2.68; p = 0.01).

**Table 4:** Results of the hierarchical logistic regression for factors associated with recent tobacco smoking among girls in private high schools (n = 1418)

	step 1	R^2^		step 2	R^2^	ΔR^2^	step 3	R^2^	ΔR^2^	step 4	R2	ΔR2
		0.04			0.08	0.04		0.11	0.07		0.21	0.10
**Parameter**	**OR**	**CI 95%**	**p**	**OR**	**CI 95%**	**p**	**OR**	**CI 95%**	**p**	**OR**	**CI 95%**	**p**
												
**Age**	1.56	(1.27-1.91)	0.00	1.61	(1.31-1.98)	0.00	1.61	(1.30-2.00)	0.00	1.73	(1.35-2.23)	0.00
***Someone you live with smoke***												
**Yes**				2.17	(1.56-3.02)	0.00	2.02	(1.41-2.90)	0.00	1.74	(1.13-2.68)	0.01
**No**				1.00	.	.	1.00	.	.	1.00	.	.
***I can easily get attention and care from my parents***												
**Never**				3.00	(1.30-6.91)	0.01	2.78	(1.13-6.83)	0.03	4.37	(1.19-16.04)	0.03
**Sometimes/rarely**				1.56	(1.12-2.19)	0.01	1.46	(1.04-2.06)	0.03	1.53	(1.06-2.20)	0.02
**Always/often**				1.00	.	.	1.00	.	.	1.00	.	.
***Do you have a religion***												
**No**							1.65	(1.07-2.56)	0.03	1.78	(1.14-2.78)	0.01
**Yes**							1.00	.	.	1.00	.	.
***Voluntary participation in religious youth meeting***												
**Not even once**							2.93	(1.60-5.34)	0.00	2.76	(1.49-5.12)	0.00
**1 to 3 times a month**							1.74	(0.80-3.78)	0.15	1.47	(0.71-3.07)	0.28
**At least once a week**							1.00	.	.	1.00	.	.
***You went out with friends at night***												
**At least once a week**										14.20	(6.64-30.37)	0.00
**1 to 3 times a month**										5.27	(2.41-11.55)	0.00
**Not even once**										1.00	.	.

For girls, not having a religion and not attending religious youth meetings were significant factors in the model. Not attending this type of activity at least once a week was associated with CS; this factor was associated with a three-fold increase in likelihood of CS (_a_OR = 2.76, 95% CI = 1.49-5.12; p < 0.001).

Girls who hang out with friends at night were 14 times more likely to smoke (_a_OR = 14.20, 95% CI = 6.64-30.37; p < 0.001).

When we evaluated the R^2 ^and ΔR^2 ^regression values for both genders, we noted that the variables that best explained smoking behavior in the model were those selected with Step 4 because the greatest ΔR^2 ^appeared between Steps 3 and 4.

## Discussion

The results of this study confirmed the importance of familial, religious and leisure factors on smoking behavior among adolescents of both genders. For both genders, older age, not having a religion, living with someone who smoked and the frequency of nightlife with friends were associated with smoking behavior. However, the familial and religious factors identified for girls were different from the ones observed for boys. For girls, receiving care and attention from parents and attending religious meetings were also inversely associated with CS. In contrast, for boys, the death of one or both parents was associated with CS.

In this study, we did not observe gender differences in the one-month CS prevalence. This result differed from studies performed at public schools in São Paulo, which suggested that the CS prevalence was higher among girls [6]. This also differs from findings in other countries, which show a higher prevalence of CS among boys [19,29]. This suggests that the CS prevalence even within the same city may vary between genders according to socio-cultural contexts.

Despite the similar prevalence of smoking in both genders, there were gender differences in factors associated with CS. Familial relationships and parental monitoring were unevenly associated with smoking by gender. Despite the finding that second-hand smoke exposure at home increased by 50-70% the likelihood of recent tobacco consumption in both genders, which is in agreement with the majority of the literature [1,11], the type of familial interaction that may influence smoking differed by gender. Perhaps, due to the characteristics of the female gender, girls found it important to be able to recognize their parents' attention and care. In this context, O'Byrne et al. [10] suggested that tobacco prevention programs aimed at adolescents must be focused on improving the relationship between parents and children. In their study with 7^th ^to 12^th^-grade adolescents, a healthy familial relationship was the main protective factor for the first use of tobacco. However, it is still not clear whether this association between familial relationship and adolescent smoking is modified by parents' use of tobacco [30] or not [31]. Nevertheless, in studies on tobacco experimentation, this association has a stronger influence on girls than boys [13].

For boys, the death of one or both parents was associated with tobacco use. This finding suggested that more important than the parent-son relationship is the need for both parents to be alive, even if they may be divorced. A study performed in 11 European countries showed that the parents marital status only influenced the initiation of smoking when divorce was rare in the country [24]. With that in mind, the only rare family structure status in our sample was the death of one or both parents (about 4% of the total sample).

Regarding religiosity, studies that sought to comprehend the role of religiosity in drug use suggested that there was a distinction among internal factors (religion membership, believing in God and the importance given to religion), as well as external factors (frequency of attendance at religious groups) [32-35]. However, the majority of the studies that sought to assess the role of this external expression of religiosity in drug use have not distinguished attendance frequency at conventional religious activities, such as services and masses, from the attendance frequency at youth religious meetings as we did [32]. In our study, the only religious factor that was associated with CS was the latter, which occurred only among girls. For both genders, not having a religion increased the odds of CS. Yet, among boys none of the internal or external religiosity variables were associated with recent CS. A possible explanation for this finding is the fact that in Brazil it is rare to openly discuss smoking during religious services, and therefore, these interactions would have added little to the adolescents' decision to smoke cigarette [36]. Nevertheless, the finding among boys is in agreement with results from a longitudinal study of 12-18 year-old British adolescents [13]. Among girls, merely having a religion did not seem to protect them from CS; instead, the network of friends in the religious group had a more protective effect because a statistical significance was found only for the religious factor that represented the public expression of religiosity in a group. This points to the group role once again as a mediator for the use or nonuse of tobacco specifically in girls. Perhaps, girls who attended adolescent religious groups had friends who did not use tobacco and who disapproved of this behavior [15].

In this context, we should highlight the important role of religion in the psychosocial development of adolescents, a domain of the developmental process that is not always included in the health literature [37]. In this developmental perspective, religiosity, family and friends merge to form a foundation for decision making: to experiment or not to experiment tobacco. Religion seems to develop as a "social controller" through its moral standards. This role places drugs, including cigarette smoking, in a category of reprehensible actions [38], in other words, actions that are not approved by "God". Gorsuch [39] posited that the Church prevents drug use by encouraging parents to supervise their children and establish anti-drug rules in their home. As in our sample we also found that parents smoking status is associated with adolescents CS, it suggests that, religiosity and family may simultaneously influence the decision of not smoking: family religiosity may be influencing adolescent religiosity and parents' religiosity may be the reason of parents not smoking. Stylianou [40] proposed a theory suggesting that the perception of immorality and personal responsibility on physical self-destruction that religions bring to their members controls these individuals' attitudes when faced with opportunities to use drugs, including tobacco.

This protective role of religion was also observed in an investigation that collected data on the education, religiosity, and moral attitudes of 16,604 individuals in 15 countries [41]. According to the authors, individual behavioral patterns and moral attitudes were more strongly oriented by religion in countries where religiosity was generally more important in social life. For example, in a cross-sectional survey among almost 13,000 adolescents in seven Latin American countries, all mainly Catholic by history, researchers identified that higher levels of religious practice behaviors were significantly associated with lower odds of initiation of tobacco smoking and lower odds of opportunities to use tobacco [12].

In both genders, hanging out with friends at night for parties was associated with CS, and this association was stronger among girls. Although participants were not questioned about the use of tobacco by their friends, our results indirectly pointed to the relevance of the frequency of hanging out with friends without parental supervision. Similarly, a North American study of African-American adolescents showed that the factor most strongly associated with smoking was the relationships with friends. When the variables describing the activities with friends were inserted in the model, all the familial factors that had previously demonstrated an association with CS were no longer significant for both genders [18]. In contrast, in Barcelona, a cross-sectional survey among high school students showed that the influence of a group on using tobacco was stronger among boys [29]. Thus, the findings corroborate the Social Developmental Theory [20] because tobacco use was associated with social influences both in terms of family members and leisure activities with friends. Even the religiosity aspects that were characterized by social activities seemed to have reinforced the anti-tobacco attitude of the adolescents [9].

Therefore, the present study suggests that preventive measures against CS among adolescents should emphasize gender peculiarities. Prevention programs that focus on family and school need to be designed separately and tailored for boys and girls in order to take into account the possible adverse risk factors and protective factors that are gender-specific. Moreover, this study highlighted the importance of reducing the adverse risk factors associated with tobacco use during adolescence to reduce the likelihood of other substances consumption [3,42].

One strategy would be to encourage parents to have a clear anti-tobacco attitude to pass on to their children from their adolescence. A recent Swedish study showed that the majority of adolescents in Sweden were receptive to their parents' anti-tobacco attitude because the practice was not based on punishments but on explanations and limits [43]. Although our analysis suggested that the use of tobacco by someone at home was a possible risk factor for tobacco use by adolescents, the literature has also shown that this factor may be reversed by communication on the topic. A study among Dutch adolescents suggested that parents who used tobacco were a risk factor for their children only when the frequency and quality of the communication between parents and children were poor [8].

In the present study, the frequency of hanging out with friends was the factor most strongly associated with CS in both genders, with evidence of a "dose-response" association. Although hanging out with friends is part of a healthy process of adolescent socialization, these results suggested that parents should be more prudent when allowing their sons and daughters to frequently hang out. And especially for girls, the additional attention and care may help to lower the likelihood of CS. In addition, parental attitudes may help to shorten the communication distance between parents and their children; parents may monitor their adolescents more closely and also become more aware of the behavior of their children's friends. Similarly, encouraging a religious connection may also play a positive role in reducing smoking among adolescents.

Since this was a cross-sectional survey, the data analyses have some limitations. For instance, the analyzed factors may have had associations with CS, but it was not possible for us to establish causal relationships. Additionally, due to the fact that a self-reported questionnaire was used, the questions were subject to interpretation by the participants. The adolescent respondents may have underreported their tobacco use [44], and smoking behaviors may have been underestimated because the study asked questions only about cigarettes. Further, we did not have data from public high schools students in São Paulo, so the data is not generalizable to the overall adolescent population in the city. The strength of this study included the inclusion of individuals rarely studied, wealthy adolescents from the largest and richest city of Brazil. Carlini-Cotrim et al. [45] have pointed out that is difficult to access private schools in Sao Paulo; despite this difficulty, were able to overcome barriers through our various telephone contacts and visits to the school principals.

## Conclusions

The results of this study may be substantially important for the programs that could influence determinants connected to gender and for building preventive models based on factors that could be more significant for either boys or girls in order to reduce CS among adolescents.

## Competing interests

The authors declare no competing interests.

## Authors' contributions

*ZMS: *wrote the manuscript and did the analyses; *SSM: *supervised the analysis and the paper writing process; *ESO: *did the literature revision and data discussion; *JSA: *critically revised the manuscript for important intellectual content; *ARN: *conceived the study, and participated in its coordination. All authors read and approved the final manuscript.

## Pre-publication history

The pre-publication history for this paper can be accessed here:

http://www.biomedcentral.com/1471-2458/10/748/prepub

## Supplementary Material

Additional file 1 **Survey questionnaire**. Click here for file
