# Supplementary material for: Adolescent gender differences in the determinants of tobacco smoking: a cross sectional survey among high school students in São Paulo
Source: BMC Public Health. 2010 Dec 3;10:748. doi: 10.1186/1471-2458-10-748 (PMC3004838; doi:10.1186/1471-2458-10-748)
Supplement: Additional file 1 — Survey questionnaire. [file 1471-2458-10-748-S1.PDF]

Questionnaire

You have been invited to participate at a survey about health behavior among students from the city of São Paulo. This questionnaire mainly brings up questions about leisure, family and the use of substances.

Don't write your name on the questionnaire because it is ANONYMOUS. It means that we are not allowed to know who answered each question.

It is very important that you read the questions and the alternatives of the answers carefully. You have just to tick an X in the answer you think it's more appropriate. There's no right or wrong answer. Answer the questions according to your experience, but all the alternatives have an answer.

It's very important that you honestly answer the questions and do not leave any unanswered or blank question. The results of this survey will be useful for the health and education professionals to better know the behavior of the young people in order to improve the quality of their services.

If you don't want to participate or answer some question, just leave it blank.

1. **Gender:** 1 Male 2 Female

2. **How old are you?** .... years-old.

3. **Tick an X on the frequency that you did the following activities last month, that is, the last 30 days:**

	**Almost every day**	**At least once a week**	**Once to three times a month**	**Never**
a) Played video game or Internet games				
b) Practiced some sports or physical activities for leisure				
c) Read books of your own free will				
d) Went out at night with friends (to parties, bars or shows)				
e) Practiced leisure activities such as, playing an instrument, singing, dancing, painting, drawing, writing				
f) Went on a walk with friends to malls, streets, squares or parks				
g) Used the Internet for fun (Orkut, MSN, songs, films)				
h) Participated at a volunteer at social work				
i) Participated at collective				

prayers (masses, cults or sessions) of your own free will				
j) Participated at meetings for youngsters of some religion of your own free will				
k) Participated at artistic activities, in some religious group, of your own free will (theater, music, entertainment, painting, etc)				

4.
A. How many days did you skip classes the last 30 days?
1 I didn't skip
2 Skipped 1 to 3 days
3 Skipped 4 to 8 days
4 Skipped 9 days or more

B. Which was the main reason for you to skip classes the last 30 days?
1 I didn't skip
2 I was sick or had a medical appointment
3 I was lazy
4 I was late or overslept
5 To go out or have fun
6 Another reason ....

5.
A. Have you ever tried some alcoholic beverages (beer, draught beer, wine, pinga, caipirinha, aperitifs, cider, and others)?
1 No
2 Yes

B. How old were you when you tried alcoholic beverages for the first time?
1 I've never tried
2 I was .... years-old
3 I don't remember

C. If you have ever tried, where were you when you drank alcoholic beverages for the first time?
1 I've never drunk
2 At home
3 A nightclub or bar
4 Friends´ or relatives´ house
5 I don't remember
6 Others ....

D. If you have ever tried, who offered you the alcoholic beverage when you drank for the first time?
1 I've never drunk
2 Nobody offered me, I bought myself
3 Relatives
4 Friends
5 I don't remember
6 Others ....

E. From one year now, that is, in the last 12 months, did you drink any alcoholic beverage?
1 No
2 Yes

F. From one month now, that is, in the last 30 days, did you drink any alcoholic beverage?
1 No
2 Yes, I drank 1 to 5 days in the month
3 Yes, I drank 6 to 19 days in the month
4 Yes, I drank 20 days or more in the month

G. If you drank any alcoholic beverage, which one did you drink in the last 30 days? (YOU CAN TICK MORE THAN ONE ANSWER)
1 I didn't drink
2 Beer or draught beer
3 Pinga
4 Whisky
5 Vodka
6 Cognac
7 Liqueur
8 Cider or champagne
9 Wine
10 Alcoholic smoothies or caipirinha
11 Ice beverages (E.g.: Smirnoff ice)
12 Others

H. Have you ever drunk any alcoholic beverage until you get drunk (get a load on)?
1 No
2 Yes

The next question is still about drinking alcoholic beverages. It's important that you answer this question thinking about how many shots of the alcoholic beverage you drank. In this question you have to consider A SHOT EQUALS TO:
1 glass of draught beer or
1 can of beer or
1 goblet of wine or
1 small glass of vodka or pinga
1 bottle of ice beverage
For example: If you drank 3 cans of beer and 2 bottles of ice beverage on the same occasion, so you drank 5 shots of alcoholic beverage.

Question:

I. From one month now, that is, in the last 30 days, how many times did you drink 5 shots or more of an alcoholic beverage on the same occasion? *(Look up at the board of shots)*

1 Never
2 Once
3 Twice
4 3 to 5 times
5 6 to 9 times
6 10 or more times
7 I don't remember

J. From one month now, that is, in the last 30 days, have you seen advertisements of alcoholic beverages on TV, magazines, newspapers, radio or Internet?

1 I haven't seen
2 Yes, I saw 1 to 5 days in the month
3 Yes, I saw 6 to 19 days in the month
4 Yes, I saw 20 days or more in the month
5 I don't know

K. Have you ever bought any alcoholic beverage (even if it was not for you)?

1 No
2 Yes
3 I've tried, but I couldn't

6.

A. Have you ever drunk any energetic beverage (Red Bull®, Flash Power®, Flying Horse®, Bad Boy®, Blue Energy®, Burn®)?

1 No
2 Yes

B. If you have ever drunk any energetic beverage, was it mixed with alcoholic beverage?

1 I've never drunk energetic beverages
2 No, I've never mixed with alcoholic beverages
3 Yes, I have mixed with alcoholic beverages

7.

A. Have you ever smoked cigarettes?

1 No
2 Yes

B. From one year now, that is, in the last 12 months, have you smoked any cigarette?

1 No
2 Yes

C. From one month now, that is, in the last 30 days, have you smoked any cigarette?

1 No
2 Yes, I've smoked 1 to 5 days in the month
3 Yes, I've smoked 6 to 19 days in the month
4 Yes, I've smoked 20 days or more in the month

D. How old were you when you smoked a cigarette for the first time?

1 I've never smoked
2 I was .... years-old
3 I don't remember

E. If you smoke, how many cigarettes do you smoke a day?
1 I don't smoke
2 1 to 10 cigarettes a day
3 11 to 20 cigarettes a day
4 More than 20 cigarettes a day

F. From one year now, that is, in the last 12 months, have you tried to quit smoking?
1 I haven't smoked in the last 12 months
2 Yes, I've tried to quit smoking and I got it
3 Yes, I've tried to quit smoking and I didn't get it
4 I haven't tried to quit

8.
A. Have you ever inhaled any product to get "high"? For example: deodorant, glue, ether, paint remover, gasoline, benzine, nail polish remover, paint thinner, nail polish, turpentine, paint.
1 No
2 Yes

B. From one year now, that is, in the last 12 months, have you inhaled any product to get "high"?
1 No
2 Yes

C. From one month now, that is, in the last 30 days, have you inhaled any product to get "high"?
1 No
2 Yes, I've inhaled 1 to 5 days in the month
3 Yes, I've inhaled 6 to 19 days in the month
4 Yes, I've inhaled 20 days or more in the month

D. If you have inhaled any product to get "high", which one of these products have you inhaled in the last 30 days? (YOU CAN TICK MORE THAN ONE ALTERNATIVE)
1 I haven't inhaled
2 Deodorant
3 Glue
4 Ether
5 Gasoline
6 Paint thinner/ turpentine/ paint or benzine
7 Nail polish/ nail polish remover
8 Others ....

E. How old were you when you inhaled these products to get "high" for the first time?
1 I've never inhaled
2 I was .... years-old
3 I don't remember

F. If you have inhaled any of these products, where did you get them the last time you inhaled?
1 I've never inhaled
2 I had in my house
3 Friends gave to me
4 I don't remember
5 Others ....

9.
A. Have you tried marijuana (or hashish)?
1 No
2 Yes

B. From one year now, that is, in the last 12 months, did you smoke marijuana?
1 No
2 Yes

C. From one month now, that is, in the last 30 days, did you smoke marijuana?
1 No
2 Yes, I’ve smoked 1 to 5 days in the month
3 Yes, I’ve smoked 6 to 19 days in the month
4 Yes, I’ve smoked 20 days or more in the month

D. How old were you when you smoked marijuana for the first time?
1 I’ve never smoked
2 I was .... years-old
3 I don’t remember

10.
A. Have you ever had any tranquilizers or sleeping pills without medical prescription? For example: Diazepam, Dienpax®, Valium®, Lorax®, Rohypnol®, Psicosedin®, Somalium®, Apraz®, Rivotril®, Alprazolam, Lexotan®, Dalmadorm®, Dormonid®, Bromazepam, Frontal®, Olcadil®. (DO NOT CONSIDER TEAS OR ANY OTHER NATURAL MEDICINES, SUCH AS MARACUJINA®)
1 No
2 Yes. What’s the name of the pill? ....

B. From one year now, that is, in the last 12 months, have you had any tranquilizers or sleeping pills without medical prescription?
1 No
2 Yes

C. From one month now, that is, in the last 30 days, have you had any tranquilizers or sleeping pills without medical prescription?
1 No
2 Yes, I’ve had 1 to 5 days in the month
3 Yes, I’ve had 6 to 19 days in the month
4 Yes, I’ve had 20 days or more in the month

D. How old were you when you had any tranquilizers or sleeping pills without medical prescription for the first time?
1 I’ve never had
2 I was .... years-old
3 I don’t remember

E. If you have ever had any tranquilizers or sleeping pills, how did you get them for the first time?
1 I’ve never had
2 There were in my house
3 Someone of my family gave to me
4 I got with friends
5 I don’t remember
6 Others ....

11. Have you ever had Holoten®, Carpinol® or Medavane® to get "high"?
1 No
2 Yes. Which one did you get the last time? ....

12.
A. Have you ever had any diet/ weight loss pills or any stay-awake pills without medical prescription? For example: Hipofagin®, Inibex®, Desobesi®, Moderine®, Absten®, Fagolipo®, Dualid®. (DO NOT CONSIDER SWEETENERS, SHAKES OR TEAS)
1 No
2 Yes. What's the name of the one you last had?...

B. From one year now, that is, in the last 12 months, have you had any diet/ weight loss pills or any stay-awake pills without medical prescription?
1 No
2 Yes

C. From one month now, that is, in the last 30 days, have you had any diet/ weight loss pills or any stay-awake pills without medical prescription?
1 No
2 Yes, I've had 1 to 5 days in the month
3 Yes, I've had 6 to 19 days in the month
4 Yes, I've had 20 days or more in the month

D. How old were you when you had any diet/ weight loss pills or any stay-awake pills without medical prescription for the first time?
1 I've never had these medicines
2 I was .... years-old
3 I don't remember

E. If you have ever had any diet/ weight loss pills or any stay-awake pills, how did you get them for the first time?
1 I've never had
2 There were in my house
3 Someone of my family gave to me
4 I got with friends
5 I bought in the night
6 I don't remember
7 Others ....

13.
A. Have you tried cocaine?
1 No
2 Yes

B. From one year now, that is, in the last 12 months, have you sniffed cocaine?
1 No
2 Yes

C. From one month now, that is, in the last 30 days, have you sniffed cocaine?
1 No
2 Yes, I've sniffed 1 to 5 days in the month
3 Yes, I've sniffed 6 to 19 days in the month
4 Yes, I've sniffed 20 days or more in the month

D. How old were you when you sniffed cocaine for the first time?
1 I've never sniffed
2 I was .... years-old
3 I don't remember

14.
A. Have you tried crack or merla?
1 No
2 Yes. Which one have you tried? ....

B. From one year now, that is, in the last 12 months, have you smoked crack or merla?
1 No
2 Yes

C. From one month now, that is, in the last 30 days, have you smoked crack or merla?
1 No
2 Yes, I've smoked 1 to 5 days in the month
3 Yes, I've smoked 6 to 19 days in the month
4 Yes, I've smoked 20 days or more in the month

D. How old were you when you smoked crack or merla for the first time?
1 I've never smoked
2 I was .... years-old
3 I don't remember

15.
A. Have you ever tried Artane®, Bentyl®, Akineton® or lily tea to get "high"?
1 No
2 Yes. Which one did you last have? ....

B. From one year now, that is, in the last 12 months, have you had Artane®, Bentyl®, Akineton® or lily tea to get "high"?
1 No
2 Yes

C. From one month now, that is, in the last 30 days, have you had Artane®, Bentyl®, Akineton® or lily tea to get "high"?
1 No
2 Yes, I've had 1 to 5 days in the month
3 Yes, I've had 6 to 19 days in the month
4 Yes, I've had 20 days or more in the month

D. If you have had Artane®, Bentyl®, Akineton® or lily tea, how old were you when you had it to get "high" for the first time?
1 I've never had
2 I was .... years-old
3 I don't remember

16. Have you ever tried heroin or opium to get "high"?
1 No
2 Yes. Which one did you last try? ....

17. Have you ever tried any of these medicines to get “high”? For example: Morfina, Tylex®, Setux®, Sylador®, Tramal® (Tramadol), Dolantina® (Meperidina ou Petidina), Fentanil®, Dolosal®, Belacodid®.
1 No
2 Yes. Which one did you last try? ....

18. Have you ever tried LSD (acid) or mushroom tea?
1 No
2 Yes. Which one have you tried? ....

19.
A. Have you tried ecstasy?
1 No
2 Yes

B. From one month now, that is, in the last 30 days, have you had ecstasy?
1 No
2 Yes, I’ve had 1 to 5 days in the month
3 Yes, I’ve had 6 to 19 days in the month
4 Yes, I’ve had 20 days or more in the month

20.
A. Have you ever tried Benflogin® to get “high”?
1 No
2 Yes

B. From one month now, that is, in the last 30 days, have you had Benflogin® to get “high”?
1 No
2 Yes, I’ve had 1 to 5 days in the month
3 Yes, I’ve had 6 to 19 days in the month
4 Yes, I’ve had 20 days or more in the month

21. Have you ever had any anabolic steroids to increase your muscle mass or your strength? For example: Anabolex®, Androlone®, Androviron®, Decadurabolin®, Durabolin®, Durateston®, Equipoise®, Parabolan®, Primobolan®.
1 No
2 Yes. Which one did you last have? ....

22. Have your ever heard of any other drug not mentioned in this questionnaire?
1 No
2 Yes, they are: ....

23. Who do you live with? (YOU CAN TICK MORE THAN ONE ANSWER)
1 Father
2 Stepfather
3 Mother
4 Stepmother
5 Brother(s) or sister(s)
6 Grandmother or Grandfather
7 Others ....

24. Your parents:
1 They live together
2 They live separated
3 One of them (or both) has already died
4 Others ....

25. Tick an X on the frequency that the following situations happened to you last month, that is, in the last 30 days:

	Always	Many times	Sometimes	Few times	Never
a) My parents or keepers defined rules about what I can do at home					
b) My parents or keepers defined rules about what I can do out of my house					
c) I easily got the attention and care from my mother and/or father or keepers					
d) I easily got money from my mother and/or father or keepers					

26. Tick an X on the frequency that, during the last 30 days, <u>your parents or keepers</u>:

	Always	Many times	Sometimes	Few times	Never
a) Asked you what you did at school					
b) Praised you when you did something well done					
c) Had some meal with you					
d) Asked where you have been and with whom					
e) Talked to you for ten minutes or more					
f) Talked to you about the problems that the alcoholic beverages can cause to young people					
g) Talked to you about the problems that other drugs can cause to young people					

The next questions are about THE OTHER PEOPLE WHO YOU LIVE WITH.

Among the people who you live with:

27. Does anyone drink alcoholic beverages (beer, draught beer, wine, pinga, caipirinha, aperitifs, cider, and others)?
1 No
2 Yes
3 I don't know

28. Does anyone drink alcoholic beverages till get drunk? (get a load on)
1 No
2 Yes
3 I don't know

29. Does anyone smoke cigarette?
1 No
2 Yes
3 I don't know

30. Does anyone have tranquilizers or sleeping pills? For example: Diazepam, Dienpax®, Valium®, Lorax®, Rohypnol®, Psicosedin®, Somalium®, Lexotan®, Rivotril®, Alprazolam, Apraz®, Bromazepam, Dalmadorm®, Dormonid®, Frontal®, Olcadil®. (DO NOT CONSIDER TEAS OR ANY OTHER NATURAL MEDICINES, SUCH AS MARACUJINA®))
1 No
2 Yes
3 I don't know

31. Does anyone have any diet/ weight loss medicine? For example: Hipofagin®, Inibex®, Desobesi®, Moderine®, Absten®, Fagolipo®, Dualid®. (DO NOT CONSIDER SWEETENER, SHAKES OR TEAS)
1 No
2 Yes
3 I don't know

32. Does anyone smoke marijuana?
1 No
2 Yes
3 I don't know

33. Does anyone sniff cocaine?
1 No
2 Yes
3 I don't know

34. Have you ever received any educational information about drugs? (YOU CAN TICK MORE THAN ONE ANSWER)
1 No
2 Yes, at my school
3 Yes, from my family
4 Yes, at my church or religious group
5 Yes, through radio or TV
6 Yes, through books or magazines
7 Yes, through the Internet
8 Yes, through friends
9 Yes, others ....

35. If you wanted to look for information about drugs, what would you do? (YOU CAN TICK MORE THAN ONE ANSWER)
1 Nothing
2 I would talk to some teacher or employee at my school
3 I would talk to someone from my family
4 I would talk to someone from my church or religious group
5 I would search information in books or magazines
6 I would search information on the Internet
7 I would talk to friends
8 I would talk to a health professional
9 Others ....

36. From one month now, that is, in the last 30 days, did you access the Internet?
1 No
2 Yes, I accessed 1 to 5 days in the month
3 Yes, I accessed 6 to 19 days in the month
4 Yes, I accessed 20 days or more in the month

37. From one month now, that is, in the last 30 days, how intense did you trust that God (or a superior power/ strength) was with you?
1 Nothing
2 A little
3 More or less
4 A lot
5 So very much

38. Do you use the beliefs (ideas) of your religion to make your decisions?
1 I don't have a religion
2 I have a religion but I don't use its ideas
3 Yes, sometimes I do
4 Yes, I always do

39. Tick an X on how much you agree or disagree with the following statements, at this moment:

	**Totally disagree**	**Disagree**	**Agree**	**Totally agree**
1. I'm optimistic about life				
2. I have short and long-range goals				
3. I feel very lonely				
4. I can see possibilities among the difficulties				
5. I have a faith that comforts me				
6. I'm afraid of my future				
7. I can remember happy and pleasurable times				
8. I feel very strong				
9. I feel I'm able to give and take love				
10. I know where I want to go				
11. I believe in the worthy of each day				
12. I feel my life is worthy and useful				

40. Marque com um "X" o quanto você concorda ou discorda com as seguintes afirmações, neste momento:

	Disagree			Neither agree nor disagree	Agree		
	**Totally**	**A lot**	**A little**		**Totally**	**A lot**	**A little**
**1**. When I make plans I go until the end	**1**	**2**	**3**	**4**	**5**	**6**	**7**
2. I'm used to dealing with problems one way or another	**1**	**2**	**3**	**4**	**5**	**6**	**7**
**3**. I'm able to depend on myself more than any other person	**1**	**2**	**3**	**4**	**5**	**6**	**7**
**4**. Keep interested on things is very important to me.	**1**	**2**	**3**	**4**	**5**	**6**	**7**
**5**. I can be on my own if I need.	**1**	**2**	**3**	**4**	**5**	**6**	**7**
**6**. I'm proud of accomplishing things in my life	**1**	**2**	**3**	**4**	**5**	**6**	**7**
**7**. I'm used to accepting things without worrying a lot.	**1**	**2**	**3**	**4**	**5**	**6**	**7**
**8**. I'm a friend of myself.	**1**	**2**	**3**	**4**	**5**	**6**	**7**
**9**. I feel I can deal with many things at the same time.	**1**	**2**	**3**	**4**	**5**	**6**	**7**
**10**. I am wayward.	**1**	**2**	**3**	**4**	**5**	**6**	**7**
**11**. I rarely think about the objective of the things	**1**	**2**	**3**	**4**	**5**	**6**	**7**
**12**. I do things one day at a time	**1**	**2**	**3**	**4**	**5**	**6**	**7**
**13**. I can face hard times because I've already experienced difficulties before.	**1**	**2**	**3**	**4**	**5**	**6**	**7**
**14**. I am disciplined.	**1**	**2**	**3**	**4**	**5**	**6**	**7**
**15**. I keep interested on things.	**1**	**2**	**3**	**4**	**5**	**6**	**7**
**16**. I can normally find a reason to laugh.	**1**	**2**	**3**	**4**	**5**	**6**	**7**
**17**. My faith in myself helps me to go through hard times.	**1**	**2**	**3**	**4**	**5**	**6**	**7**
**18**. In case of emergency, I'm a person other people can count on.	**1**	**2**	**3**	**4**	**5**	**6**	**7**
**19**. I can usually look at a situation through many ways.	**1**	**2**	**3**	**4**	**5**	**6**	**7**
**20**. Sometimes I obey myself to do things, wanting or not.	**1**	**2**	**3**	**4**	**5**	**6**	**7**
**21**. My life has a meaning.	**1**	**2**	**3**	**4**	**5**	**6**	**7**
**22**. I don't insist on things I can do nothing about.	**1**	**2**	**3**	**4**	**5**	**6**	**7**
**23**. When I am on a bad	**1**	**2**	**3**	**4**	**5**	**6**	**7**

situation I usually find the way out.							
**24**. I have enough energy to do what I have to do.	**1**	**2**	**3**	**4**	**5**	**6**	**7**
**25**. I'm ok if there are people who don't like me.	**1**	**2**	**3**	**4**	**5**	**6**	**7**

41. From one month now, that is, in the last 30 days, have you ridden a motorcycle <u>without a helmet</u>?
1 No
2 Yes

42. From one month now, that is, in the last 30 days, have you carried any king of weapon with you (E.g.: gun, knife or a wood stick)?
1 No
2 Yes

43. From one month now, that is, in the last 30 days, have you been involved in a fight with physical aggression?
1 No
2 Yes

44. From one month now, that is, in the last 30 days, have you had sexual intercourse without the condom?
1 No
2 Yes

45. From one month now, that is, in the last 30 days, have you ever had any diet/ weight loss pills with the intention to lose or control your weight?
1 No
2 Yes

46. Educational level of the breadwinner/ responsible for the family:
1 Never studied/ illiterate
2 Incomplete elementary school
3 Complete elementary school, but incomplete junior high
4 Complete junior high
5 Incomplete high school
6 Complete high school
7 Went to college, but didn't finish
8 Finished college
9 I don't know

47. In your house, there is:

A. Color TV? (Not broken)
1 No
2 Yes. How many? ....

B. Videocassete? (Not broken)
1 No
2 Yes. How many? ....

C. DVD set? (Not broken)
1 No
2 Yes. How many? ....

D. Radio? (Not broken)
1 No
2 Yes. How many? ....

E. Bathroom?
1 No
2 Yes. How many? ....

F. Cars? (Not broken)
1 No
2 Yes. How many? ....

G. Employee who works everyday for your family? E.g.: housekeeper/ maid, babysitter, driver, gardener, etc.
1 No
2 Yes. How many? ....

H. Washing machine? (Not broken)
1 No
2 Yes. How many? ....

I. Refrigerator? (Not broken)
1 No
2 Yes. How many? ....

J. Freezer (in the refrigerator or independent)? (Not broken)
1 No
2 Yes. How many? ....

SEE IF YOU HAVEN´T LEFT ANY UNANSWERED OR BLANK QUESTION.

If you want, use the space provided below to make comments.
